# The Polymorphism and Expression of EGFL7 and miR-126 Are Associated With NSCLC Susceptibility

**DOI:** 10.3389/fonc.2022.772405

**Published:** 2022-04-14

**Authors:** Weipeng Liu, Yunyun Zhang, Fengdan Huang, Qianli Ma, Chuanyin Li, Shuyuan Liu, Yan Liang, Li Shi, Yufeng Yao

**Affiliations:** ^1^ Institute of Medical Biology, Chinese Academy of Medical Sciences and Peking Union Medical College, Kunming, China; ^2^ Graduate School of Yunnan University, Yunnan University, Kunming, China; ^3^ Department of Thoracic Surgery, The 3rd Affiliated Hospital of Kunming Medical University, Kunming, China

**Keywords:** EGFL7, miR-126, NSCLC, DNA methylation, SNPs

## Abstract

Previous investigations have reported that microRNA-126 (miR-126) and its host gene, epidermal growth factor-like domain-containing protein 7 (EGFL7) are involved in lung cancer progression, suggesting EGFL7 and miR-126 play a joint role in lung cancer development. In this study, we analyzed the methylation-associated regulation of EGFL7 and miR-126 in non-small cell lung cancer (NSCLC) and further investigated the association between EGFL7/miR-126 polymorphisms and NSCLC susceptibility in the Han Chinese population. Based on our data, relative to those in adjacent normal tissue, both EGFL7 expression and miR-126 expression were decreased significantly in lung cancer tissue (*P* = 3x10^-4^ and *P* < 1x10^-4^), and the expression of EGFL7 mRNA and miR-126 was significantly correlated in both NSCLC tissue n = 46, r = 0.43, P = 0.003 and adjacent normal tissue n = 46, r = 0.37, P = 0.011. Differential methylation analysis indicated that methylation levels of multiple CG loci in EGFL7 were significantly higher in the lung cancer samples than in the normal samples (*P* < 0.01). Moreover, EGFL7 mRNA and miR-126 were significantly upregulated after treatment with the DNA demethylating agent 5-aza-2′-deoxycytidine (5-Aza-CdR) in lung cancer cell lines. In addition, the A allele of rs2297538 was significantly associated with a decreased NSCLC risk (OR = 0.68, 95% CI: 0.52~0.88), and the expression of EGFL7 and miR-126 was significantly lower in rs2297538 homozygous G/G tumor tissue than in A/G+A/A tumor tissue (*P* = 0.01 and *P* = 0.002). Our findings suggest that the expression of EGFL7 and miR-126 in NSCLC can be concomitantly downregulated through methylation and the EGFL7/miR-126 polymorphism rs2297538 is correlated with NSCLC risk. Together, these results provide new insights into the pathogenesis of NSCLC.

## Introduction

Lung cancer is one of the most common cancers in the world; in 2020, there were more than 2.2 million newly reported lung cancer cases and over 1.79 million new lung cancer-related deaths worldwide (1). Non-small cell lung cancer (NSCLC) is the most prevalent lung cancer type, accounting for 85–90% of all lung cancer cases (2).

Recently, studies have demonstrated that genetic factors also play important roles in the occurrence of lung cancer, and twin studies have shown that the heritability of lung cancer is approximately 18% (3, 4). In recent years, a number of differentially expressed genes have been identified in lung cancer (5–7), and many researchers have turned their attention to the study of common single nucleotide polymorphisms (SNPs) in human carcinogenic/anticancer genes (8–10). These studies can not only expand our understanding of the pathogenesis of lung cancer but also provide new clues for the diagnosis and treatment of this disease.

Epidermal growth factor-like domain-containing protein 7 (EGFL7) is a secreted protein that was initially reported to play a key role in angiogenesis (11, 12). Subsequently, the dysregulation of EGFL7 has been found in a variety of tumors, including lung cancer (13), hepatocellular carcinoma (14), acute myeloid leukemia (15) and malignant pleural mesothelioma (16), suggesting that EGFL7 participates in tumorigenesis through a wide range of effects.

MicroRNA−126 (miR−126) is located within intron 7 of EGFL7 on human chromosome 9q34.3 (17). Recent studies have shown that miR-126 is downregulated in various cancer tissues, including breast cancer (18), pancreatic cancer (19) and lung cancer (20). In recent years, increasing evidence has demonstrated the role of miR−126 in lung tumorigenesis *via* targeting multiple genes including CCR1 (21), PIK3R2 (22), Crk (23) and so forth. In addition, miR−126 has been found to play a role in both diagnosis and prognosis of NSCLC (24, 25), thus indicating that miR−126 could be a promising biomarker in lung cancer.

Previous investigations have shown that miR−126 is not transcribed from its own promoter but is likely transcribed together with its host gene EGFL7 (17), indicating that the expression levels of miR-126 and EGFL7 may be regulated by the same mechanisms during the occurrence of NSCLC. Furthermore, studies have identified CpG islands around the transcription initiation site of EGFL7 (17); thus, it is reasonable that miR-126 and EGFL7 can be silenced by methylation of cytosine residues. In addition, recent studies have revealed that single nucleotide polymorphisms (SNPs) in gene regulatory and coding regions could confer risk of lung cancer by regulating the expression of specific genes (26–28). Thus, SNPs in the transcriptional regulatory region and coding region of EGFL7 might also play a role in the regulation of EGFL7 and miR-126 expression and are further involved in NSCLC susceptibility. Herein, we explored the methylation-associated regulation of miR-126 and EGFL7 in NSCLC and further investigated the association between EGFL7 polymorphisms and NSCLC susceptibility in the Han Chinese population. We showed that the expression of miR-126 and EGFL7 is concomitantly downregulated in NSCLC through methylation and that the eQTL-missense polymorphism of EGFL7 is associated with lung cancer risk in a Han Chinese population.

## Materials and Methods

### Subjects and Tissue Samples

This study was approved by the Ethics Committee of the Third Affiliated Hospital of Kunming Medical University, and all study protocols were performed in accordance with the Declaration of Helsinki. Written informed consent was acquired from each participant.

We performed a case-control association study, a total of 497 patients with NSCLC treated in the Third Affiliated Hospital of Kunming Medical University in Yunnan were selected as the case group, and 502 healthy people undergoing a physical examination in the hospital during the same period were selected as the control group. The NSCLC patients were diagnosed according to the Chinese Medical Association guidelines for clinical diagnosis and treatment of lung cancer (Edition 2018) at the Third Affiliated Hospital of Kunming Medical University. The lung cancer histological type and pathologic stage were identified according to the International System for Staging Lung Cancer (29). The NSCLC patient inclusion criteria: 1) the patients were histologically and pathologically diagnosed NSCLC (adenocarcinoma and squamous cell carcinoma); 2) the patients had not received chemotherapy and radiotherapy. The criteria for the exclusion was 1) the patients with a prior history of primary cancer other than lung cancer; 2) the patients with small cell lung cancer or unclear pathological diagnosis; 3) the patient with malignant tumors except lung cancer.

A total of 46 matched sets of primary NSCLC tumors and adjacent normal tissues were acquired from NSCLC patients at the Third Affiliated Hospital of Kunming Medical University. All tissues were identified by pathological examination and fresh-frozen at -80°C.

### Quantitative RT–PCR

Total RNA was isolated from tissues or cells using TRIzol reagent. The PrimeScript™ RT reagent Kit with gDNA Eraser (TaKaRa Bio Inc, Tokyo, Japan) was used to synthesize cDNA. We reverse transcribed 1 µg of total RNA, and diluted cDNA at a final concentration of 20 µg/µL. Quantitative real-time PCR was carried out under the following conditions using SYBR Green to detect the expression levels of EGFL7 and miR-126 in NSCLC tissues and corresponding adjacent tissues in clinical patients: denaturation at 95°C for 10 min, followed by 40 cycles of denaturation at 95°C for 15 s, annealing at 60°C for 15 s, and extension at 72°C for 15 s. GAPDH was used as the internal control of EGFL7, and U6 was used as the internal control of miR-126. All the primers are listed in **Table S1**.

### Cell Culture

All the cell lines used in this study were originally obtained from the ATCC. Human lung epithelial BEAS-2B cells were cultured in Dulbecco’s modified Eagle’s medium (DMEM, Gibco) containing 10% fetal bovine serum (FBS, Gibco), SPC-A1 cells were cultured in RPMI-1640 with 10% fetal bovine serum (FBS, Gibco), H1299 cells were cultured in Dulbecco’s modified Eagle’s medium (DMEM, Gibco) containing 10% fetal bovine serum (FBS, Gibco). Cells were cultured in a cell incubator at 37°C with 5% CO2.

### 5-Aza-2’-Deoxycytidine (5-Aza-CdR) Treatment

5-Aza-CdR has been widely used to demonstrate the correlation between loss of methylation in specific gene regions and activation of the associated genes (30). In the current study, BEAS-2B, SPC-A1 and H1299 cells were seeded in six-well culture dishes 24 h prior to treatment with 5-Aza-CdR. 5-Aza-CdR was continuously administered by replacing the medium containing 5-Aza-CdR (0 μM and 10 μM) every 24 h for 2 days. The dose of 5-Aza-CdR (10 μM) was chosen based on our preliminary studies. Similar reactivation was shown of EGFL7 and miR-126 expression when cells were treated with varying concentrations (1–10 μM) of 5-Aza-CdR as well as previously published studies (31, 32). Cells were then harvested for total RNA extraction to demonstrate whether treatment with the demethylating agent was able to increase EGFL7 and miR-126 mRNA expression in these cell lines.

### SNP Selection

In this study, 1670 bp upstream of the EGFL7/miR-126 transcription start site was chosen as the promoter region according to a previous study (33). JASPAR (http://jaspar.genereg.net/) was used to predict whether the SNPs in the promoter region of EGFL7 are located in the transcription factor binding site and disrupt the binding of specific transcription factors (34). Missense variants with a minor allele frequency (MAF) greater than 0.05 were called and filtered using the Ensembl Variant Effect Predictor (http://www.ensembl.org/vep) (35). Finally, rs1332793, rs9411260 and rs2297538 were chosen as candidate SNPs in the current study.

### DNA Extraction and Sequencing

A heparin anticoagulant tube was used to collect 10 ml of venous blood from the study subjects. DNA was extracted by a QIAamp DNA Blood Mini Kit (Qiagen, Hilden, Germany), and the concentration and purity of the genomic DNA were detected by a Multiskan Skyhigh full-wavelength enzyme plate (ND-2000, Thermo Fisher Scientific). After genomic DNA was extracted, it was stored in a -20°C refrigerator for later use.

Three SNPs rs1332793, rs9411260 and rs2297538 were genotyped by TaqMan probe real-time fluorescence quantitative polymerase chain reaction (RTFQ-PCR). The probes and primers were designed and produced by Thermo Fisher Scientific Company (Waltham, MA, USA), and TaqMan Genotyping Master Mix was purchased from ABI. PCR amplification was carried out in 384-well reaction plates with 2.5 μL Master Mix, 0.125 μL primer and probe (FAM and VIC) mix, 1.375 μL ddH2O and 1 μL genomic DNA in each well. Amplification was conducted in a QuantStudio 6 Flex Fast Real-Time PCR system as follows: 95°C preheat denaturing for 10 min, 92°C for 10 s and 60°C for 1 min, all repeated for 40 cycles. We also confirmed the genotyping results of 20 randomly selected individuals using Sanger sequencing, and no genotyping errors were found.

### In-Silico Analysis of EGFL7 Expression and Methylation Status

GEPIA was used to explore the relative expression of EGFL7 in lung cancer tissue and normal tissue (http://gepia.cancer-pku.cn/), which provides the expression data of tumors and normal samples from the TCGA and GTEx projects (36). SurvivalMeth was used to explore the methylation status of EGFL7 in lung cancer tumor tissues and normal tissues (http://bio-bigdata.hrbmu.edu.cn/survivalmeth) (37).

### Statistical Analysis

GraphPad Prism 8.3.0 software was used for statistical analysis; Hardy-Weinberg equilibrium was used to test sample representativeness. Student’s t-test was used to analyze the age difference between the NSCLC group and the control group. The χ2 test was used to analyze whether there was a sex difference between the NSCLC group and the control group. The differences in allele frequencies and genotype distribution of rs1332793, rs9411260, and rs2297538 in the NSCLC group and control groups were also analyzed *via* χ2 test, and the significance threshold was set at P<0.017 (0.05/3). SHEsis online software (38) was used to analyze the linkage disequilibrium between SNPs, and D’ > 0.8 indicates strong linkage between SNPs. The genetic pattern of SNPs was analyzed by SnpStats online software (39), obtaining the optimal genetic pattern according to the AIC (akaike information criterion) and BIC (bayesian information criterion) values. Relative expression levels of EGFL7 and miR-126 are presented as the means of 2^−ΔΔCt^. Statistical tests against different groups were conducted using a two-tailed t-test. Spearman’s correlation analysis was performed to detect the correlation between EGFL7 and miR-126 expression in NSCLC tumor tissue and adjacent normal tissue. The significance threshold was set at P<0.05. Statistical analyses were performed using SPSS 21 (Chicago, IL) and GraphPad Prism 7.00.

## Results

### Expression of miR-126 and EGFL7 mRNA Is Significantly Reduced in NSCLC Tissues Compared With Adjacent Normal Tissues

**Figure 1:**
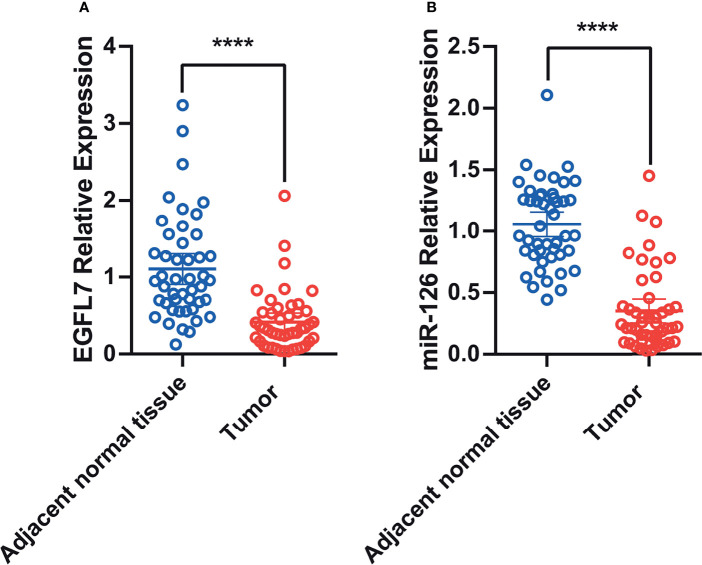
Scatter plot of EGFL7 mRNA **(A)** and miR-126 **(B)** expression in lung tumor tissue and adjacent normal tissues. The values on Y-axis were presented as 2^–ΔΔCt^. **** represent P ≤ 0.0001.

Previous studies have reported low miR-126 expression in lung cancer tissue (20). In silico analysis of EGFL7 mRNA expression indicated that the expression of EGFL7 in lung cancer tissue was lower than that in normal lung tissue (*P* < 0.01, **Figure S1**). To validate these results, we tested miR-126 and EGFL7 mRNA expression in lung cancer tissue along with matched adjacent normal tissue from 46 NSCLC patients using qRT–PCR. We found that EGFL7 and miR-126 expression decreased significantly in lung cancer tissue compared with adjacent normal tissue (*P* = 3x10^-4^ and *P* < 1x10^-4^, respectively, **Figures 1A, B**), which was consistent with in silico analysis (**Figure S1**) and previous results (20).

### Expression of miR-126 and EGFL7 mRNA Is Correlated in NSCLC Tissues and Adjacent Normal Tissues

**Figure 2:**
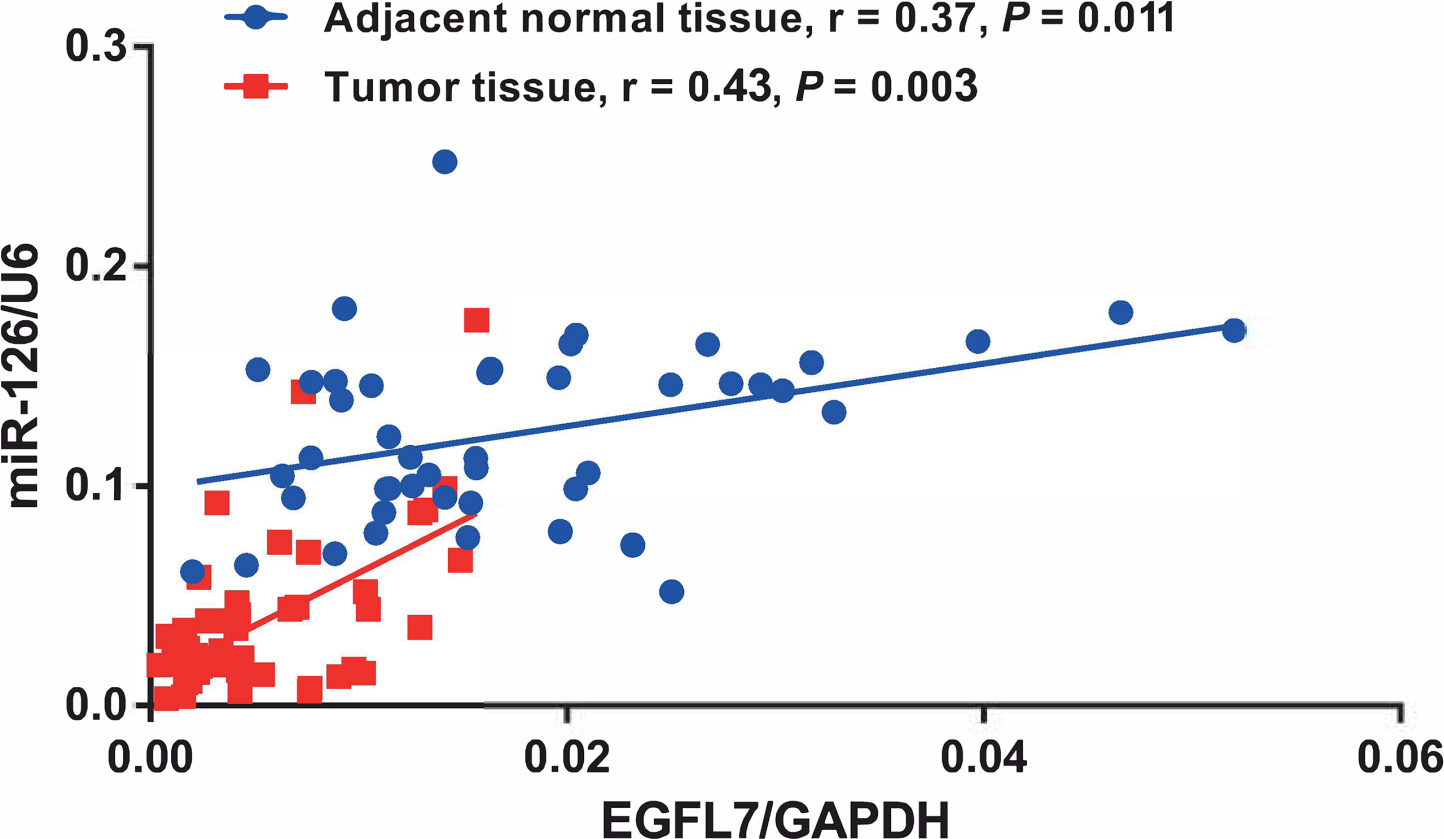
Correlation between EGFL7 and miR-126 expression in NSCLC tumor tissue and adjacent normal tissue. Spearman’s correlation coefficient (r) and *P value*s are shown for each analysis.

To further investigate whether miR-126 and EGFL7 were concomitantly expressed in NSCLC as previously described for prostate cancer (17) and malignant pleural mesothelioma (16), we performed spearman’s correlation analysis to detect the correlation of miR-126 and EGFL7 expression in both NSCLC tissues and adjacent normal tissues. Our results showed that the expression of EGFL7 and miR-126 were significantly correlated in both NSCLC tissues (n = 46, r = 0.43, *P* = 0.003, **Figure 2**) and adjacent normal tissues (n = 46, r = 0.37, *P* = 0.011, **Figure 2**).

### EGFL7 and miR-126 Are Upregulated After Treatment With the DNA Demethylating Agent 5-aza-2’-Deoxycytidine (5-Aza-CdR) in Lung Cancer Cell Lines

Previous studies have reported CpG islands in EGFL7 (17). To investigate the methylation status of EGFL7 in lung cancer tumor tissues and normal tissues, we performed the differential methylation analysis of EGFL7 CG loci in patients with adenocarcinoma (AC) and squamous cell carcinoma (SCC) using the SurvivalMeth database (http://bio-bigdata.hrbmu.edu.cn/survivalmeth). Our analysis revealed that the methylation levels of 4 CG loci (cg08529852, cg14548542, cg17443080 and cg20997159) within EGFL7 were significantly higher in the AC samples than in the normal samples (*P* < 0.01, **Figure S2**). Furthermore, the methylation levels of 6 CG loci (cg04074066, cg05936059, cg08529852, cg14353956, cg14548542 and cg20997159) were significantly higher in the SCC samples than in the normal samples, while the methylation level of cg21184800 markedly decreased in SCC tumor samples (*P* < 0.01, **Figure S2**). These results were consistent with the result that EGFL7 mRNA expression decreased in lung tumor tissue and indicated that the expression of EGFL7 mRNA in NSCLC can be downregulated through methylation.

**Figure 3:**
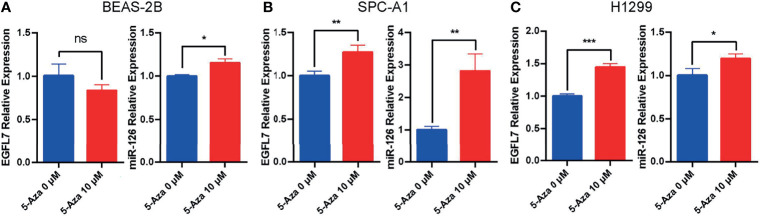
Expression of EGFL7 and miR-126 after demethylating agent 5-Aza treatment in BEAS-2B **(A)**, SPC-A1 **(B)** and H1299 **(C)** cell lines. * represents P ≤ 0.05, ** represents P ≤ 0.01, *** represents P ≤ 0.001, ns represents P > 0.05.

To verify the expression of miR-126 and EGFL7 can be silenced by methylation of cytosine residues in lung cancer cells. BEAS-2B (non-tumorigenic human bronchial epithelial cells), SPC-A1 and H1299 cells were treated with 5-Aza-CdR (10 μM) for 48 h. After treatment with the DNA demethylating agent 5-Aza-CdR, EGFL7 mRNA and miR-126 were significantly upregulated in both SPC-A1 and H1299 lung cancer cell lines (*P* < 0.05, **Figure 3**), while no significant difference of EGFL7 mRNA expression was observed in BEAS-2B (**Figure 3**). Overall, our results indicated that the expression of miR-126 and EGFL7 mRNA in NSCLC can be concomitantly regulated through methylation.

### Association of EGFL7 and miR-126 Gene SNPs With NSCLC

Recent studies have revealed that single nucleotide polymorphisms (SNPs) in gene regulatory and coding regions could confer risk of lung cancer by regulating the expression of specific genes (26–28), hinting that SNPs in the transcriptional regulatory region and coding region of EGFL7 might also play a role in the regulation of EGFL7 and miR-126 expression, and be further involved in NSCLC susceptibility. Hence, in the current study, we investigated the association between EGFL7 polymorphisms and NSCLC susceptibility in the Han Chinese population.

A total of 502 healthy control samples and 497 NSCLC samples were collected in this study. The clinical characteristics of the study subjects are summarized in **Table 1**. No significant difference in age or sex was found between the control and NSCLC groups (*P* = 0.09 and *P* = 0.13, respectively) (**Table 1**). In the NSCLC group, 338 patients had adenocarcinoma (AC), 159 patients had squamous cell carcinoma (SCC), 204 patients were Clinical stage I+ II, and 293 patients were Clinical stage III+ IV.

**Table 1:** Characteristics of the subjects enrolled in the current study.

	NSCLC	Control	*P* value
N	497	502	
Ages (years)	55.99 ± 10.77	54.70 ± 13.19	0.09
Sex (M/F)	325/172	305/197	0.13
AC	338		
SCC	159		
Clinical stage			
I	124		
II	80		
III	158		
IV	135		

NSCLC, non-small cell lung cancer; AC, adenocarcinoma; SCC, squamous cell carcinoma.

All three SNPs (rs1332793, rs9411260 and rs2297538) were in Hardy-Weinberg equilibrium (HWE) in the control and NSCLC groups (P > 0.05). The allelic and genotypic distributions of these three SNPs among the healthy control and NSCLC groups are presented in **Table 2**. Among these SNPs, the allele and genotype distributions of rs2297538 were significantly different between the control and NSCLC groups (P < 0.017). The A allele of rs2297538 might be associated with a decreased risk of NSCLC (*P* = 0.003; OR = 0.68, 95% CI: 0.52~0.88; **Table 2**). In addition, the GG genotype frequency of rs2297538 was higher in the NSCLC group than in the control group (*P* = 0.012; **Table 2**). We further evaluated the distributions of the alleles and genotypes of these 3 SNPs in different NSCLC pathological types (AC and SCC) and different NSCLC pathological stages (I+II and III+IV). Logistic regression analysis revealed no significant differences in genotypic and allelic distributions of these SNPs among different subgroups (**Table S2**). The AIC and BIC were calculated to evaluate the best fitting inheritance model (codominant, dominant, recessive, overdominant and log‐additive) for each SNP in this study (39). Only rs2297538 genotypes were found to be associated with NSCLC risk (**Table 3**). The best fit inheritance model with the lowest AIC and BIC for rs2297538 was dominant, and the A/G and A/A genotypes conferred a greater risk of NSCLC (*P* = 3.1x10^-3^; OR = 1.62, 95% CI: 0.65–4.01). These results indicate that EGFL7 and miR-126 play roles in NSCLC pathogenesis.

**Table 2:** The allelic and genotypic distribution of SNPs in EGFL7 genes among healthy control and NSCLC groups.

SNPs	Control	NSCLC	Control VS NSCLC	
			*P* value	OR[95%CI]
rs1332793				
C	333(0.332)	291(0.293)	0.061	0.83 [0.69~1.01]
T	671(0.668)	703(0.707)		
C/C	54(0.108)	42(0.085)	0.169	
C/T	225(0.448)	207(0.416)		
T/T	223(0.444)	248(0.499)		
rs9411260				
G	209(0.208)	187(0.188)	0.261	0.88 [0.71 ~1.10]
A	795(0.792)	807(0.812)		
G/G	25(0.050)	21(0.042)	0.541	
A/G	159(0.317)	145(0.292)		
A/A	318(0.633)	331(0.666)		
rs2297538				
A	157(0.156)	111(0.112)	0.003	0.68 [0.52~0.88]
G	847(0.844)	883(0.888)		
A/A	12(0.024)	8(0.016)	0.012	
A/G	133(0.265)	95(0.191)		
G/G	357(0.711)	394(0.793)		

The statistical significant threshold was set at P<0.017 (0.05/n, n= 3) after Bonferroni correction.

NSCLC, non-small cell lung cancer.

**Table 3:** Inheritance model analysis of SNPs in the EGFL7 gene between healthy controls and NSCLC patients.

SNPs	Models	Genotypes	Control	NSCLC	OR(95%CI)	*P* value	AIC	BIC
		T/T	223 (44.4%)	248 (49.9%)	1			
	Codominant	C/T	225 (44.8%)	207 (41.6%)	1.21 (0.93-1.58)	0.170	1388.4	1408
		C/C	54 (10.8%)	42 (8.4%)	1.42 (0.91-2.21)			
	Dominant	T/T	223 (44.4%)	248 (49.9%)	1	0.080	1386.8	1401.6
rs1332793		C/T-C/C	279 (55.6%)	249 (50.1%)	1.25 (0.97-1.60)			
	Recessive	T/T-C/T	448 (89.2%)	455 (91.5%)	1	0.230	1388.5	1403.2
		C/C	54 (10.8%)	42 (8.4%)	1.30 (0.85-1.98)			
	Overdominant	T/T-C/C	277 (55.2%)	290 (58.4%)	1	0.290	1388.8	1403.5
		C/T	225 (44.8%)	207 (41.6%)	1.14 (0.89-1.47)			
	Log-additive	—	—	—	1.20 (0.99-1.45)	0.060	1386.4	1401.1
		A/A	318 (63.4%)	332 (66.8%)	1			
	Codominant	G/A	159 (31.7%)	144 (29%)	1.16 (0.88-1.52)	0.510	1390.5	1410.2
		G/G	25 (5%)	21 (4.2%)	1.23 (0.67-2.24)			
	Dominant	A/A	318 (63.4%)	332 (66.8%)	1	0.250	1388.6	1403.3
rs9411260		G/A-G/G	184 (36.6%)	165 (33.2%)	1.16(0.90-1.51)			
	Recessive	A/A-G/A	477 (95%)	476 (95.8%)	1	0.600	1389.6	1404.3
		G/G	25 (5%)	21 (4.2%)	1.17 (0.65-2.13)			
	Overdominant	A/A-G/G	343 (68.3%)	353 (71%)	1	0.340	1389	1403.7
		G/A	159 (31.7%)	144 (29%)	1.14 (0.87-1.49)			
	Log-additive	—	—	—	1.13 (0.91-1.41)	0.250	1388.6	1403.3
		G/G	357 (71.1%)	394 (79.3%)	1			
	Codominant	A/G	133 (26.5%)	95 (19.1%)	1.54 (1.14-2.08)	1.2 x 10^-2^	1383.1	1402.7
		A/A	12 (2.4%)	8 (1.6%)	1.62 (0.65-4.01)			
	Dominant	A/G-A/A	357 (71.1%)	394 (79.3%)	1	3.1x10^-3^	1381.1	1395.8
rs2297538		C/T-C/C	145 (28.9%)	103 (20.7%)	1.55 (1.16-2.07)			
	Recessive	G/G-A/G	490 (97.6%)	489 (98.4%)	1	0.410	1389.2	1403.9
		A/A	12 (2.4%)	8 (1.6%)	1.46 (0.59-3.62)			
	Overdominant	G/G-A/A	369 (73.5%)	402 (80.9%)	1	5.6 x10^-3^	1382.2	1396.9
		A/G	133 (26.5%)	95 (19.1%)	1.52 (1.13-2.05)			
	Log-additive	—	—	—	1.46 (1.13-1.90)	4 x10^-3^	1381.6	1396.3

The statistical significant threshold was set at P<0.017 (0.05/n, n= 3) after Bonferroni correction.

NSCLC, non-small cell lung cancer; AIC, akaike information criterion; BIC, bayesian information criterion.

### Rs2297538 Is Associated With miR-126 and EGFL7 mRNA Expression

**Figure 4:**
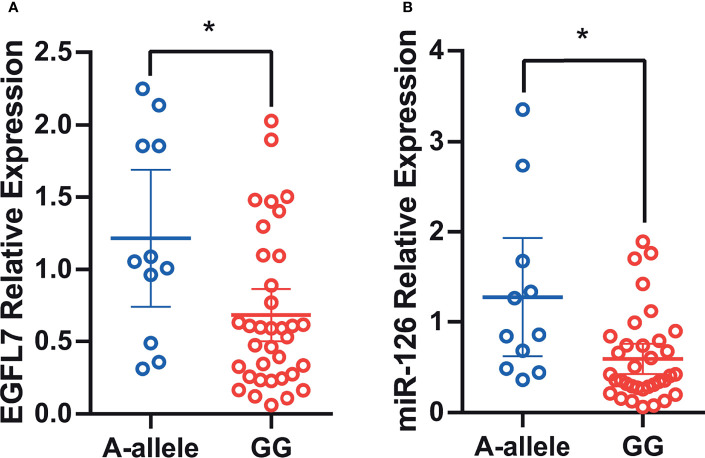
Association of rs2297538 with EGFL7 mRNA **(A)** and miR-126 **(B)** expression in lung tumor tissue (N = 46). The values on Y-axis were presented as 2^–ΔΔCt^. * represents P ≥ 0.05.

Single nucleotide polymorphisms (SNPs) in gene regulatory and coding regions often confer a risk of lung cancer by affecting gene expression (26–28). To determine whether rs2297538 was related to the expression of nearby genes, we conducted eQTL analysis between this SNP and miR-126 and EGFL7 mRNA expression using qRT–PCR in 46 lung cancer tissues. We compared the mRNA expression of EGFL7 and miR-126 between the risk allele homozygous group [G/G] and the other genotypic groups [A/G + A/A], and we found that the expression of EGFL7 and miR-126 was significantly lower in the G/G group (lung cancer risk allele homozygotes) than in the A/G+A/A group (*P* = 0.01 and *P* = 0.002, respectively, **Figures 4A, B**). These results suggested that reduced expression of EGFL7 and miR-126 might be risk factors for NSCLC and that rs2297538 may confer a risk of NSCLC by regulating EGFL7 and miR-26 expression.

## Discussion

In the current study, we found that the expression of miR-126 and EGFL7 mRNA was concomitantly downregulated through the methylation of CpG islands in NSCLC and that the eQTL-missense polymorphism of EGFL7, rs2297538 (located at 386 bp 5’ of miR-126), was significantly associated with the risk of NSCLC in the Han Chinese population.

Several studies have reported aberrant expression of EGFL7 and miR-126 in various human cancers (40), suggesting that EGFL7 and miR-126 play a joint role in cancer development. We found that miR-126 and EGFL7 are significantly downregulated in lung cancer tissues, which is consistent with the results of Fan and Yang et al. (13, 20). Our results indicated that aberrant expression of EGFL7 and miR-126 could play an important role in the development of NSCLC. One of the reasons for the association between aberrant expression of EGFL7 and miR-126 and NSCLC could be the change in DNA hypermethylation.

Recently, reduced EGFL7 and miR-126 expression regulated *via* DNA hypermethylation of the promoter region was observed in ovarian cancer (41), prostate cancer (17) and malignant pleural mesothelioma (16). However, only a few studies have shown the same regulatory mechanism in lung cancer. For example, Watanabe et al. showed miR-126 can be silenced by the DNA methylation of its host gene EGFL7 in lung cancer cell lines (42).Our results also showed that the expression of miR-126 and EGFL7 mRNA in NSCLC is concomitantly regulated, probably by methylation. This is in accordance with findings in other cancer types, including malignant pleural mesothelioma (MPM) (16), breast cancer (13), prostate cancer (17) and lung cancer (42). In 2011, Azhikina et al. reported that the methylation level of the EGFL7 promoter is higher in lung tumors than in healthy lung tissue (43), which further supports our results. We thus further confirmed that the *EGFL7* promoter CpG island is highly methylated and thus downregulates EGFL7 and miR-126 in lung tumors.

In addition to hypermethylation of the promoter region, polymorphisms could play an important role in the development of NSCLC. For example, our previous study found that another common SNP, rs4636297, located in the EGFL7 gene region (which is also located in the pri-miR-126 gene) was associated with susceptibility to cervical cancer (44), and a previous study revealed a significant relationship between the EGFL7 3’UTR variant rs1051851 and the overall survival of metastatic colorectal cancer patients (45), suggesting that EGFL7 and miR-126 SNPs might be associated with multiple cancer types. In the current study, we found that rs2297538, which located in exon 7 of EGFL7 and changes valine to isoleucine, was significantly associated with the risk of NSCLC. To our knowledge, this is the first study to demonstrate an association between EGFL7 and miR-126 genetic variants and NSCLC. Notably, in a recent study, Duan et al. found that individuals carrying rs2297538 homozygous GG had lower leukocyte mitochondrial DNA copy numbers in the polycyclic aromatic hydrocarbon exposure group (46). As a lower mitochondrial DNA copy number could predict an increased risk of cancer induced by polycyclic aromatic hydrocarbon exposure (46) and miR-126 has been reported to regulate mitochondrial energy metabolism (47, 48), it is reasonable to hypothesize that rs2297538 may confer a risk of NSCLC by regulating miR-126 expression and further affecting mitochondrial functions. However, because the function of rs2297538 is currently unknown, we need to expand the sample size for further validation and explore its role in miR-126 and EGFL7 mRNA expression as well as mitochondrial function.

In the current study, we firstly found that rs2297538 was associated with the expression of miR-126 and EGFL7 mRNA in lung cancer tissues. As majority of previous studies focus on transcriptional regulation of EGFL7/miR-126 by DNA methylation in multiple tumor cell lines, our brand new finding in this article suggested that EGFL7 and miR-126 mRNA levels could be regulated *via* DNA methylation as well as single nucleotide polymorphisms. Moreover, the risk rs2297538 G allele might be correlated with lower expression of miR-126 and EGFL7 mRNA in lung cancer tissues, which indicated that reduced expression of miR-126 and EGFL7 might be risk factors for NSCLC. However, increased expression of EGFL7 has been reported in certain epithelial cancers (13). This may be somewhat surprising, given that elevated expression of EGFL7 may play a crucial role in cancer biology by modulating tumor angiogenesis (40). One of the reasons for the discrepancy might be the different types of cancer, as the lung is inherently a highly vascularized tissue and maintains high levels of EGFL7. In addition, decreased expression of EGFL7 may confer a risk of NSCLC *via* other biological mechanisms, such as altering the mitochondrial function of lung cancer cells. Considering that heterogeneity plays a key role in cancer management, we also performed the EGFL7 expression assay at single cells’ level using the human protein atlas data base (49, 50). Our results revealed that EGFL7 expressed mainly in endothelial cells and alveolar type 2 cells. In 2016, Pinte et al. reported that EGFL7 was able to repress endothelial cells activation (51). Their finding indicated EGFL7 could play a role in tumoral angiogenesis and tumor progression. In addition, alveolar type 2 cells were reported to play an active role in enhancing alveolar fluid clearance and reducing lung inflammation. Thus, the alveolar type 2 cells therapy was used and it was reported to have great potential effects for acute lung injury/acute respiratory distress syndrome in several preclinical studies (52). Based on these evidences, altered expression of EGFL7 in endothelial cells and alveolar type 2 cells could be associated with the lung cancer susceptibility. 

Notably, there are several limitations in the current study, and we are cautious in the interpretation of the present results. Our data indicated that methylation levels of several CG loci in *EGFL7* were significantly higher in the lung cancer samples than in the normal samples and 5-Aza-CdR treatment of NSCLC cell lines could result in up-regulation of EGFL7 mRNA expression. However, the classic locus-specific methylation experiments to explore the methylation frequency of CpG-islands EGFL7 in NSCLC cell lines and the methylation changes in different pathological types and stages should be carried out in the future. In addition, even though our results indicated that expression of miR-126 and EGFL7 mRNA are significantly reduced in NSCLC tissues compared with adjacent normal tissues, the protein expression difference of EGFL7 also needs to be investigated.

In summary, we confirmed that the expression of EGFL7 and miR-126 in NSCLC can be concomitantly downregulated through methylation of CpG islands. We also report that *EGFL7* and *miR-126* are correlated with NSCLC risk in the Han Chinese population, and our results suggest that rs2297538 may confer a risk of NSCLC by altering the gene expression of EGFL7 and miR-126. Together, these results provide new insights into the pathogenesis of NSCLC.

## Data Availability Statement

The original contributions presented in the study are included in the article/**Supplementary Material**. Further inquiries can be directed to the corresponding authors.

## Ethics Statement

The studies involving human participants were reviewed and approved by The Ethics Committee of the Third Affiliated Hospital of Kunming Medical University. The patients/participants provided their written informed consent to participate in this study.

## Author Contributions

YY, LS, and WL designed the study and interpreted the results. WL, YZ, FH, CL, and SL conducted the SNP genotyping, the primary functional assays, including qRT-PCR, cell line experiments, and analysis of those data. QM and YL contributed to collection of clinical samples. YY and LS drafted the manuscript. All authors contributed to the final version of the paper.

## Funding

This work was supported by grants from the Fundamental Research Funds for the Central Universities and the PUMC Youth Fund (3332021071), the National Science Foundation for Young Scientists of China (82103190), the Special Funds for High-level Healthy Talents of Yunnan Province (L-201615, H-2018014 and D-2018037). The funders had no role in study design, data collection and analysis, decision to publish or preparation of the manuscript.

## Conflict of Interest

The authors declare that the research was conducted in the absence of any commercial or financial relationships that could be construed as a potential conflict of interest.

## Publisher’s Note

All claims expressed in this article are solely those of the authors and do not necessarily represent those of their affiliated organizations, or those of the publisher, the editors and the reviewers. Any product that may be evaluated in this article, or claim that may be made by its manufacturer, is not guaranteed or endorsed by the publisher.

## Supplementary Material

Supplementary Figure 1 The relative expression of EGFL7 in lung cancer tissue and normal tissue from GEPIA database. N, normal; T, tumor; AC, adenocarcinoma; SCC, squamous.

Supplementary Figure 2 The methylation status of EGFL7 in lung cancer tumor tissues and normal tissues from SurvivalMeth database. AC, adenocarcinoma; SCC, squamous.
