# Supplementary figures and images for: The Polymorphism and Expression of EGFL7 and miR-126 Are Associated With NSCLC Susceptibility
Source: Front Oncol. 2022 Apr 14;12:772405. doi: 10.3389/fonc.2022.772405 (PMC9046731; doi:10.3389/fonc.2022.772405)

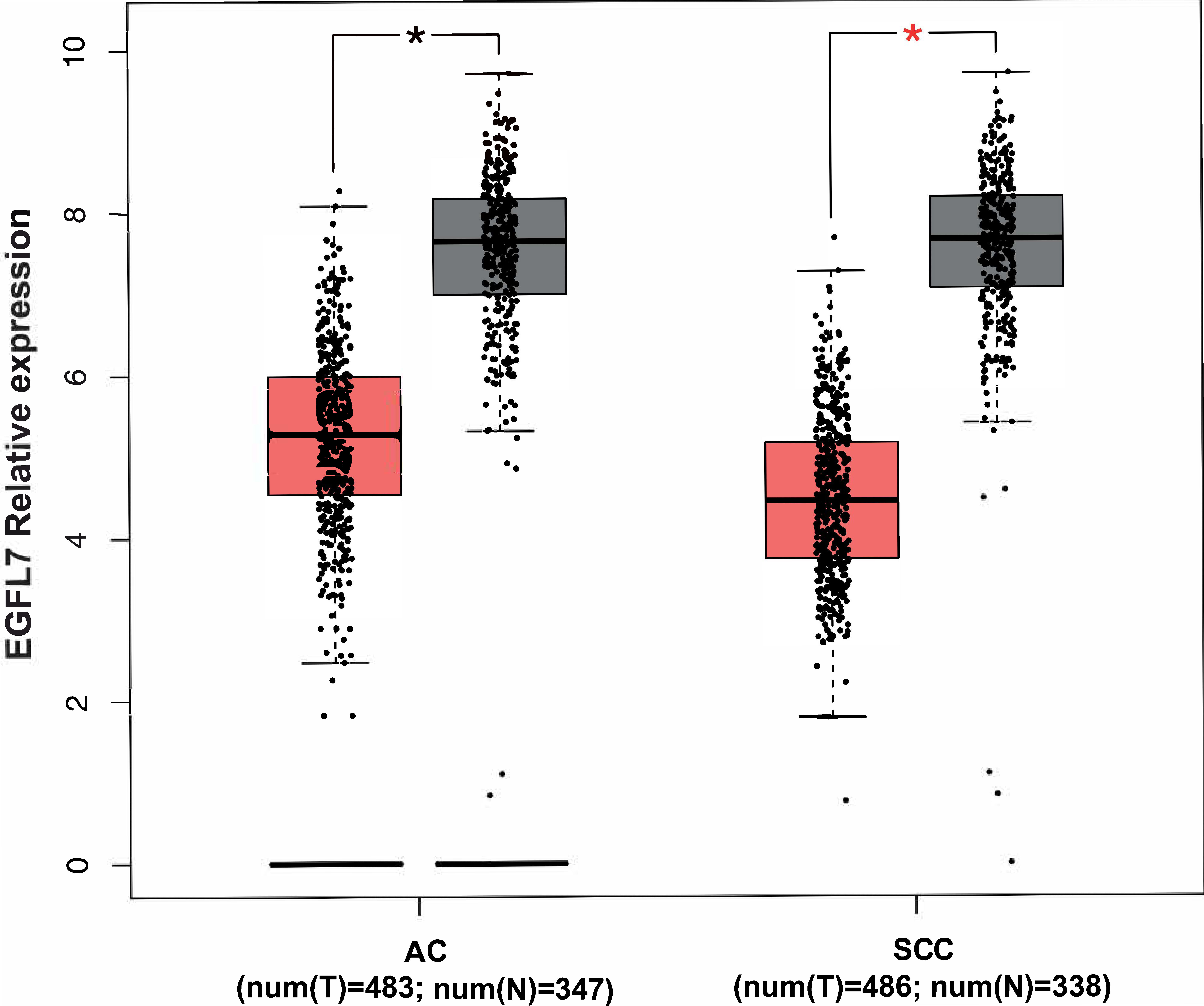

Supplement: Supplementary Figure 1 — The relative expression of EGFL7 in lung cancer tissue and normal tissue from GEPIA database. N, normal; T, tumor; AC, adenocarcinoma; SCC, squamous. [file Image_1.jpeg]
