# Supplementary material for: The Polymorphism and Expression of EGFL7 and miR-126 Are Associated With NSCLC Susceptibility
Source: Front Oncol. 2022 Apr 14;12:772405. doi: 10.3389/fonc.2022.772405 (PMC9046731; doi:10.3389/fonc.2022.772405)
Supplement: Supplementary file 3 [file Table_1.docx]

| **Gene** | **Primer** | **Sequence (5’-3’)** |
| --- | --- | --- |
| *EGFL7* | Forward | TGAATGCAGTGCTAGGAGGG |
|  | Reverse | GCACACAGAGTGTACCGTCT |
| *miR-126* | Forward | CACGCATCGTACCGTGAGTAAT |
|  | Reverse | CCAGTGCAGGGTCCGAGGTAT |
| *GAPDH* | Forward | GGAGCGAGATCCCTCCAAAAT |
|  | Reverse | GGCTGTTGTCATACTTCTCATGG |
| *U6* | Forward | CTCGCTTCGGCAGCACA |
|  | Reverse | AACGCTTCACGAATTTGCGT |

**TableS1. qPCR primers used in current study**

**TableS2. The Allelic and genotypic distribution of SNPs in EGFL7among NSCLC different pathological types and different NSCLC pathological stages**

| **SNPS** | **AC** | **SCC** | **AC VS SCC** | | **I+II** | **III+IV** | **I+II VS III+IV** | |
| --- | --- | --- | --- | --- | --- | --- | --- | --- |
|  |  |  | **P value** | **OR[95%CI]** |  |  | **P value** | **OR[95%CI]** |
| rs1332793 |  |  |  |  |  |  |  |  |
| C | 194(0.287) | 97(0.305) | 0.560 | 1.09  [0.82 ~1.46] | 124(0.304) | 167(0.285) | 0.519 | 0.91  [0.69~1.20] |
| T | 482(0.713) | 221(0.695) |  |  | 284(0.696) | 419(0.715) |  |  |
| C/C | 25(0.074) | 17(0.107) | 0.444 |  | 21(0.103) | 21(0.072) | 0.454 |  |
| C/T | 144(0.426) | 63(0.396) |  |  | 82(0.402) | 125(0.427) |  |  |
| T/T | 169(0.500) | 79(0.497) |  |  | 101(0.495 | 147(0.502) |  |  |
| rs9411260 |  |  |  |  |  |  |  |  |
| G | 131(0.194) | 56(0.176) | 0.506 | 0.89 [0.63~1.26] | 82(0.201) | 105(0.179) | 0.387 | 0.87  [0.63~1.20] |
| A | 545(0.806) | 262(0.824) |  |  | 326(0.799) | 481(0.821) |  |  |
| G/G | 15(0.044) | 6(0.038) | 0.807 |  | 8(0.039) | 13(0.044) | 0.427 |  |
| A/G | 101(0.299) | 44(0.277) |  |  | 66(0.324) | 79(0.270) |  |  |
| A/A | 222(0.657) | 109(0.686) |  |  | 130(0.637) | 201(0.686) |  |  |
| rs2297538 |  |  |  |  |  |  |  |  |
| A | 82(0.121) | 29(0.091) | 0.160 | 0.73  [0.47~1.14] | 51(0.125) | 60(0.102) | 0.266 | 0.80  [0.54~1.19] |
| G | 594(0.879) | 289(0.909) |  |  | 357(0.875) | 526(0.898) |  |  |
| A/A | 6(0.018) | 2(0.013) | 0.368 |  | 6(0.029) | 2(0.007) | 0.143 |  |
| A/G | 70(0.207) | 25(0.157) |  |  | 39(0.191) | 56(0.191) |  |  |
| G/G | 262(0.775) | 132(0.830) |  |  | 159(0.779) | 235(0.802) |  |  |

**Note: The statistical significant threshold was set at *P* < 0.0167 (0.05/n, n= 3) after Bonferroni correction.**

**Abbreviation: NSCLC, non-small cell lung cancer.**
